# Cardiac Troponin T (*TNNT2*) Mutations in Chinese Dilated Cardiomyopathy Patients

**DOI:** 10.1155/2014/907360

**Published:** 2014-07-07

**Authors:** Xiaoping Li, Rong Luo, Haiyong Gu, Yun Deng, Xiaolei Xu, Xiushan Wu, Wei Hua

**Affiliations:** ^1^Cardiac Arrhythmia Center, Cardiovascular Institute and Fuwai Hospital, Chinese Academy of Medical Sciences, Peking Union Medical College, Beijing 100037, China; ^2^Department of Cardiology, Sichuan Academy of Medical Sciences and Sichuan Provincial People's Hospital, Chengdu, Sichuan 610072, China; ^3^The Center of Heart Development, Key Lab of MOE for Development Biology and Protein Chemistry, College of Life Science, Hunan Normal University, Changsha, Hunan 410081, China; ^4^Department of Cardiothoracic Surgery, Cardiovascular Institute and Fuwai Hospital, Chinese Academy of Medical Sciences, Peking Union Medical College, Beijing 100037, China; ^5^Division of Cardiovascular Diseases, Mayo Clinic College of Medicine, Rochester, MN 55905, USA

## Abstract

*Background*. Dilated cardiomyopathy (DCM) is one of the leading causes of heart failure with high morbidity and mortality. Although more than 40 genes have been reported to cause DCM, the role of genetic testing in clinical practice is not well defined. Mutations in the troponin T (*TNNT2*) gene represent an important subset of known disease-causing mutations associated with DCM. Therefore, the aim of the present study was to determine the genetic variations in *TNNT2* and the associations of those variations with DCM in Chinese patients. *Methods*. An approximately 4 kb fragment of the *TNNT2* gene was isolated from 103 DCM patients and 192 healthy controls and was analyzed by DNA sequence analysis for genetic variations. *Results*. A total of 6 *TNNT2* mutations were identified in 99 patients, including a G321T missense mutation (Leu84Phe) and 5 novel intronic mutations. Alleles of two novel SNPs (c.192 + 353 C>A, OR = 0.095, 95% CI: 0.013–0.714, *P* = 0.022; c.192 + 463 G>A, OR = 0.090, 95% CI: 0.012–0.675, *P* = 0.019) and SNP rs3729843 (OR = 1.889, 95% CI: 1.252–2.852; *P* = 0.002) were significantly correlated with DCM. *Conclusions*. These results suggest that the missense mutation (Leu84Phe) and two novel SNPs (c.192 + 353 C>A, c.192 + 463 G>A) in *TNNT2* gene might be associated with DCM in the Chinese population.

## 1. Background

Idiopathic or primary dilated cardiomyopathy (DCM) is one of the leading causes of heart failure with high morbidity and mortality [1, 2]. The prevalence of DCM is 36.5 cases per 100,000 individuals, and 30–50% of all cases are diagnosed as a familial form of DCM [2–4]. Recent studies have reported that more than 40 genes, including 2 X-linked genes, are associated with DCM [5, 6]. In the vast majority of cases, these genes encode for sarcomeric contractile proteins such as troponin T (*TNNT2*), troponin I (*TNNI3*), and cardiac *α*-actin (ACTC) [7, 8].

The* TNNT2* gene (OMIM number ∗191045) encodes the thin-filament contractile protein cardiac troponin T, which links the troponin complex to tropomyosin in the sarcomere [9].* TNNT2* contains 16 exons, is located on chromosome 1q32, and comprises 25 kb of the genome. Recent data have indicated that* TNNT2* mutations are associated with DCM and that the overall frequency of* TNNT2* mutations in familial DCM is approximately 3–6% [10, 11]. In our laboratory, we found a significant association between tagging SNPs rs3729547, rs3729843 of* TNNT2* and DCM in the Chinese Han population [12]. In the present study, we further explored additional variants in DCM patients by sequencing an approximately 4 kb (3992 bp) DNA fragment covering the rs3729843 and rs3729547 mutations. We identified 6 novel* TNNT2* mutations in DCM patients, including a G12026T (c.252 G>T) missense mutation (Leu84Phe), 5 novel mutations in introns, and 2 novel single-nucleotide polymorphisms (SNP) (c.192 + 353 C>A, c.192 + 463 G>A).

## 2. Materials and Methods

### 2.1. Subjects and Selection of Tagging SNPs

This case-control study enrolled 103 unrelated DCM patients from the Fuwai Hospital. The clinical diagnosis of DCM was made in accordance with revised criteria [2]. Primary DCM was defined as systolic dysfunction (left ventricular (LV) ejection fraction <50%) with or without LV dilation in the absence of an apparent secondary cause of cardiomyopathy (such as congenital heart disease, ischemic heart disease, uncontrolled hypertension, significant valvular disease, glycogen storage disease, arrhythmogenic right ventricular cardiomyopathy, or concomitant complex congenital heart disease). Coronaroangiography was performed in all patients and patients with coronary artery disease were excluded. A total of 192 healthy, unrelated individuals from a routine health survey were enrolled as controls. This study was approved by the Ethics Committee of Fuwai Hospital. Subjects were informed of the study aims and provided written, informed consent prior to participating.

### 2.2. Samples

Blood samples were collected via puncture of the cubital vein. Tubes with ethylene diamine tetra acetic acid were used for DNA analysis. Blood samples were stored at 4°C and DNA isolation was performed by a modified salting out procedure [13] within a week of collection.

### 2.3. DNA Sequencing

Based on the SNP and linkage disequilibrium analysis of* TNNT2* in our recent study in DCM patients [12], an approximately 4-kilo-base (kb) fragment of* TNNT2* located at chr.201333582-201337484 was selected, and the fragment included exons 6, 7, 8, 9, and 10 and introns 5, 6, 7, 8, 9, and 10. The* TNNT2* fragment was amplified by PCR with primers (primer sequences available on request). Products were then sequenced using an ABI 3730 DNA Sequencer (Applied Biosystems, Foster City, CA). The DNA sequence was viewed and analyzed using the Sequencher computer program (Gene Codes Corporation, Ann Arbor, MI).

### 2.4. Statistical Analysis

Differences in the distributions of selected variables and* TNNT2* genotypes between cases and controls were evaluated using the chi-square (*χ* ^2^) test and continuous variables were analyzed using the independent-samples Student's* t*-test. The correlation between the* TNNT2* genotype and the risk of DCM was estimated by using logistic regression analysis to compute odds ratios (ORs) and 95% confidence intervals (CIs). The *χ* ^2^ test was used to determine Hardy-Weinberg equilibrium among control subjects. All statistical analyses were performed with SPSS 16.0 (SPSS Inc., Chicago, IL, USA) and a *P* value of <0.05 was considered statistically significant.

## 3. Results

### 3.1. Characteristics of the Study Cohort

The study included 295 subjects, consisting of 103 patients with DCM and 192 healthy control subjects; 4 patients and 3 control subjects were excluded from analyses due to indeterminate sequence results. A total of 79.4% of control subjects were males with a mean age of 54.0 ± 3.6 years. Patients with DCM were of a similar age to controls (51.6 ± 12.0 years; *t* = 1.700, *P* = 0.092) and were made up of a similar percentage of males (77.3%; *χ* ^2^ = 0.160, *P* = 0.689). In addition, 43.3% of DCM patients were smokers. There were no pregnant or peripartum subjects in this study. DCM patients were defined as having systolic dysfunction (LV ejection fraction <50%), LV dilation detected by echocardiogram, and no apparent secondary cause of cardiomyopathy. In DCM patients, the mean LV ejection fraction was 32.0 ± 8.4%, LV diameter was 67.7 ± 8.6 mm, and left atrium diameter was 42.7 ± 7.6 mm. The genotypic frequencies of each SNP in control subjects fit the assumption of Hardy-Weinberg equilibrium (Table 1).

### 3.2. Identification of Mutations

**Figure 1:**
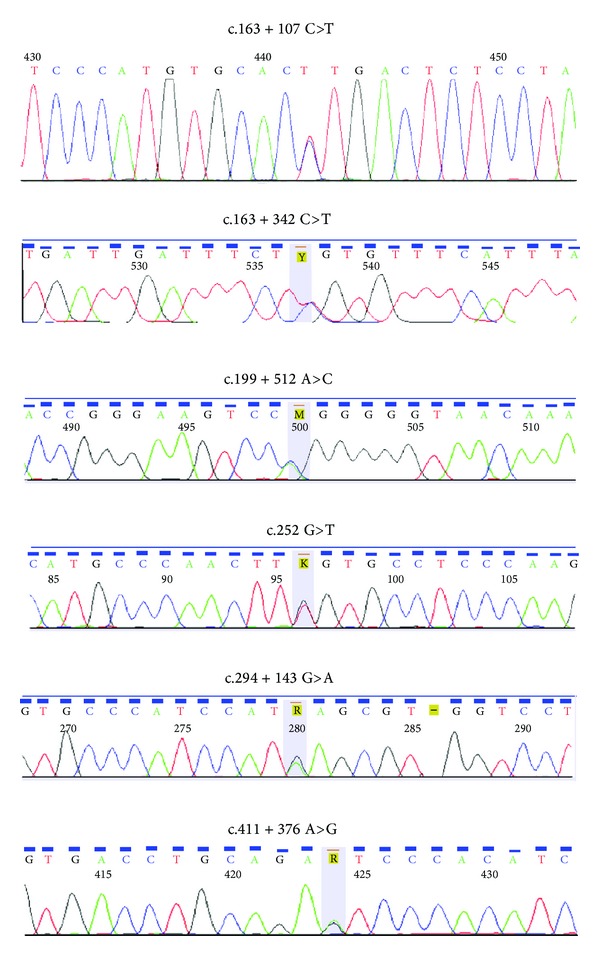
Sequences of TNNT2. Six novel variants in TNNT2 gene in DCM patients, including a missense mutation (G12026T, c.252 G>T, Leu84Phe), five novel variants in introns, respectively.

The screening of the 3992-nucleotide fragment of* TNNT2* led to the identification of one novel missense mutation. A single-nucleotide variant consisting of a G>T transversion (TTG>TTT) at nucleotide 12026 (G12026T, c.252 G>T) (Figure 1) in exon 9 was found in one DCM patient and would substitute a phenylalanine for the normal leucine at residue 84 (Leu84Phe). This patient was diagnosed at the age of 43 and was clinically symptomatic of heart failure, with LV 60 mm and LV ejection fraction 31%. Additionally, this patient had a familial history of coronary heart disease. In addition, we also identified 5 mutations in introns 6, 7, 9, and 10 in a subset of the DCM patients (Figure 1). Their clinical data are shown in Table 2.

### 3.3. Identification of Polymorphisms

During sequence analysis, 11 known SNPs and two novel SNPs (c.192 + 353 C>A, c.192 + 463 G>A) were identified in both DCM patients and control samples. We compared the genotype and allele frequencies of the* TNNT2* SNPs between the DCM patients and control subjects. Our results showed that the allele of the two novel SNPs (c.192 + 353 C>A, OR = 0.095, 95% CI = 0.013–0.714, *P* = 0.022; c.192 + 463 G>A, OR = 0.090, 95% CI = 0.012–0.675, *P* = 0.019) and SNP rs3729843 (OR = 1.889, 95% CI = 1.252–2.852, *P* = 0.002) were significantly correlated with DCM. The allele and genotype frequencies of the 13 SNPs identified in both DCM patients and control subjects are shown in Table 3, with corresponding results from statistical analysis.

## 4. Discussion

In our study, we analyzed a 3992-nucleotide sequence of the* TNNT2* gene containing exons 6, 7, 8, 9, and 10 and introns 5, 6, 7, 8, 9, 10, and 11. We identified 6 novel* TNNT2* mutations in DCM patients, including a missense mutation (G12026T, c.252 G>T, Leu84Phe) and 5 novel intronic mutations. We also found that the genotype of two novel single-nucleotide polymorphisms (c.192 + 353 C>A, c.192 + 463 G>A) and the previously identified rs3729843 [12] were correlated with DCM. These data indicate that* TNNT2* variants are associated with DCM in Chinese population. To our knowledge, the two novel SNPs and the six mutations, in which five are in intron and one is in exon, have not been reported in dilated cardiomyopathy before.

Dilated cardiomyopathy is an important cause of heart failure and the leading indication for cardiac transplantation worldwide [14]. With careful evaluation of relatives, familial disease can be identified in approximately 30% of patients with idiopathic DCM [15]. Genetic characterization of DCM has been a challenging task owing to incomplete knowledge of the genes involved in the disease as well as difficulties in determining the clinical significance of DNA variants identified in patients. More than 40 different genes have been implicated in the pathophysiology of DCM [5, 6], and* TNNT2* mutations in familial DCM are approximately 3–6% [10, 11].

Cardiac troponin (cTn) is made up of three distinct subunits, each named according to its function: cardiac troponin I (cTnI) can inhibit the actomyosin ATPase activity independently of the other Tn subunits; cardiac troponin C (cTnC) binds Ca^2+^ to a low affinity Ca^2+^-specific binding site, relieving the cTnI inhibition; and cardiac troponin T (cTnT) binds the entire cTn complex to tropomyosin (Tm) [16, 17]. Recent studies have suggested that cardiac TnT is essential not only for the structural integrity of the troponin complex but also for sarcomere assembly and cardiac contractility [18]. The troponin complex is a calcium sensor that regulates the contraction of striated muscle and modulates actomyosin ATPase activity and force [19]. Over the past decade, mutations in the* TNNT2* gene have been found to be associated with familial HCM and DCM [3, 10, 11, 20–22]. Many studies in reconstituted systems have provided valuable information on the functional effects of disease-associated mutations in TnT [23–28].

Independent laboratories have consistently observed that changes in Ca^2+^-sensitivity, ATPase activity, or force development due to cTn mutations decrease in DCM [23, 24]. All DCM mutations in cTnT (R131W, R141W, R205L, and ΔK210) showed decreases in ATPase activation [25–28]. Mutant transgenic mice expressing cTnT I79N show increased Ca^2+^ sensitivity of force development and decreased force per cross-sectional area [24]. The observed decrease in force per cross-sectional area by this mutation has been reported by different groups [29, 30]. Lin and colleagues [31] demonstrated that transgenic mice carrying either cTnT mutations F110I or R278C displayed significant increases in energy cost. Alterations in the energy cost could be explained as an increase in the rate of dissociation of myosin cross bridges and/or a decrease in the average force per cross bridge. The functional effects of a cTnT mutation (ΔK210) linked to DCM have been explored in reconstituted systems and in animal models. In vitro studies showed that cTnT ΔK210 exchanged in skinned porcine cardiac fibers decreased the relative maximal force [27, 29].

In the present study, we found a missense mutation (G12026T, c.252 G>T, Leu84Phe) in one DCM patient. This mutation was first reported in DCM patients and resulted in the substitution of a phenylalanine for the normal leucine at residue 84 in TnT. We used the website http://www.proteinmodelportal.org/query/pdb to query the crystal structure of the 52 kDa domain of human TNNT2; however, the crystal structure around the 84th amino acid in the protein is not reported up to now. Therefore, we estimate the damage of TNNT2 Leu 84 Phe transition using website http://genetics.bwh.harvard.edu/pph/. The result indicated that the mutation is predicted to be probably damaging with a score of 0.992 (total score is equal to one; sensitivity 0.7; specificity 0.97). The 84th amino acid in TNNT2 is a very conserved site in human, rat, mice, and zebrafish. The 84th amino acid was substituted by phenylalanine which function and structure with a benzene ring is different from leucine. Therefore, this mutation might contribute to the occurrence of dilated cardiomyopathy.

In addition, we found 5 intronic mutations in DCM patients as well as a significant association between the genotypes of 2 novel SNPs and rs3729843 with DCM. Although there were no encoding amino acid changes in these mutations and polymorphisms, numerous functional roles for introns have been elucidated; these functions include augmenting proteome diversity by enabling alternative splicing, enhancing gene expression, and harboring various* cis-* and* trans*-regulatory elements [32–37]. In addition, some studies also showed that some polymorphisms in noncoding regions may influence the expression level of the encoded protein, thus resulting in a more subtle variation in the associated phenotype [38]. These results suggest that* TNNT2* mutations and polymorphisms may play an important role in DCM in the Chinese Han population. However, additional functional analyses are needed to confirm the role of these variants in DCM pathogenesis.

In conclusion, based on the sequence analysis of* TNNT2* in DCM patients, the present study identified 6 novel* TNNT2* variants, a missense mutation (G12026T, c.252 G>T, Leu84Phe), 5 mutations in introns, SNP rs3729843, and 2 novel single-nucleotide polymorphisms (c.192+353 C>A, c.192+463 G>A). These TNNT2 variants might be associated with DCM in the Chinese population.

## 5. Limitations of the Study

There are clear limitations to the feasibility and practicality of screening for mutations in only one gene. Genetic analysis as performed in this study, which is limited to only a small number of potentially involved exons, does not seem to be applicable. It was impossible to screen all other genes associated with DCM, so we cannot exclude the possibility that some patients were compound heterozygotes. Functional analyses of mutations are required to confirm the findings in this study. In addition, most of patients and many SNPs are the same as those in the previous study [12] although the detecting method is different.

## Figures and Tables

**Table 1:** The sequences of the primers used to amplify the 4 kb region in *TNNT2*.

Markers	Sequences of PCR primers	Tm	Length
TNNT2-1F	CATGTTCTGTGGTGCCAGAC	57	899
TNNT2-1R	TGCCACCAAGTTCTGTCCTC	58	
TNNT2-2F	AGGAGGCTGAAGGTAAGGAT	55	691
TNNT2-2R	ACACTCACGCAGTGTGGAAC	56	
TNNT2-3F	AGGCCTTGTCACTGTGAAGC	58	573
TNNT2-3R	TGCACGATTGGTGATGGAGT	59	
TNNT2-4F	TGTGTACTGCACAAGCGTCTC	59	514
TNNT2-4R	GTGCACAAGAGGCCAGGAAG	61	
TNNT2-5F	ATAGGCATGGCGGCTTCA	60	862
TNNT2-5R	ACAGCCACCGCTTACATCAA	59	
TNNT2-6F	GGCAGTGCTGGAAGATTCTC	57	629
TNNT2-6R	GGCCATCAGAGAATGTTAGG	55	
TNNT2-7F	AATGAGTTGCAGGCGCTGAT	61	454
TNNT2-7R	CTCCAGCCAGAGCAGCATGT	61	
TNNT2-8F	CCTTGACTGCCAGAGCTGAG	58	559
TNNT2-8R	GAGAAGGTGACATCGCAGGTA	59	

**Table 2:** Mutations in *TNNT2* in DCM patients.

Markers	Exon/intron	Location of nucleotide change	Amino acid change	Number of patients	Gender	Age at diagnosis (years)	Family history	LVEDD (mm)	LVEF (%)
c.163 + 107	Intron 6	201337183 C>T	Noncoding	1	Male	37	No	82	18
c.163 + 342	Intron 6	201336948 C>T	Noncoding	1	Male	30	No	81	31
c.199 + 512	Intron 7	201336387 A>C	Noncoding	1	Male	59	Suspensive coronary heart disease	60	45
c.252	Exon 9	201334780 G>T	Leu 84 Phe	1	Male	43	Suspensive coronary heart disease	60	31
c.294 + 143	Intron 9	201334595 G>A	Noncoding	1	Male	45	No	58	40
c.411 + 376	Intron 10	201333948 A>G	Noncoding	1	Male	62	Suspensive cardiovascular disease	80	36

Note: LVEDD: left ventricular end-diastolic diameter; LVEF: left ventricular ejection fraction.

**Table 3:** Genotype and allele frequencies of the SNPs in *TNNT2* in the DCM patients and control subjects.

Marker	Genotype			*χ* ^2^, *P* value	Allele		*P* value	OR (95% CI)	Power
rs45576939	A/A	A/G	G/G		A	G	0.173	0.235 (0.029, 1.889)	28%
Patients	0 (0)	1 (0.01)	97 (0.99)	*χ* ^2^ = 2.231	1 (0.01)	195 (0.99)			
Controls	0 (0)	8 (0.043)	179 (0.957)	*P* = 0.135	8 (0.021)	366 (0.979)			
rs3729842	C/C	C/T	T/T		C	T	0.865	1.042 (0.651, 1.667)	5.4%
Patients	74 (0.755)	16 (0.163)	8 (0.082)	*χ* ^2^ = 4.282	164 (0.837)	32 (0.163)			
Controls	135 (0.722)	45 (0.241)	7 (0.037)	*P* = 0.118	315 (0.842)	59 (0.158)			
rs3729843	A/A	A/G	G/G		A	G	***0.002***	1.889 (1.252, 2.852)	85.2%
Patients	11 (0.112)	33 (0.337)	54 (0.551)	*χ* ^2^ = 8.315	55 (0.281)	141 (0.719)			
Controls	7 (0.037)	50 (0.267)	130 (0.695)	***P* = 0.004**	64 (0.171)	310 (0.829)			
rs1892028	A/A	A/G	G/G		A	G	0.140	0.770 (0.544, 1.090)	31.3%
Patients	31 (0.313)	54 (0.5)	14 (0.143)	*χ* ^2^ = 4.719	116 (0.586)	82 (0.414)			
Controls	55 (0.294)	86 (0.460)	47 (0.251)	*P* = 0.094	196 (0.521)	180 (0.479)			
rs10800775	C/C	C/T	T/T		C	T	0.152	0.766 (0.533, 1.103)	29.7%
Patients	46 (0.465)	45 (0.455)	9 (0.090)	*χ* ^2^ = 2.142	137 (0.685)	63 (0.315)			
Controls	73 (0.388)	89 (0.473)	26 (0.138)	*P* = 0.343	235 (0.625)	141 (0.375)			
rs7544061	T/T	T/C	C/C		T	C	0.612	0.821 (0.383, 1.761)	73%
Patients	0 (0)	10 (0.101)	89 (0.899)	*χ* ^2^ = 0.274	10 (0.051)	188 (0.949)			
Controls	0 (0)	23 (0.116)	166 (0.834)	*P* = 0.601	23 (0.061)	355 (0.939)			
rs3767545	A/A	A/G	G/G		A	G	0.294	1.680 (0.638, 4.424)	20.3%
Patients	0 (0)	8 (0.081)	91 (0.919)	*χ* ^2^ = 1.160	8 (0.04)	190 (0.960)			
Controls	0 (0)	9 (0.049)	175 (0.951)	*P* = 0.282	9 (0.024)	359 (0.976)			
rs3729547	C/C	T/C	T/T		C	T	***0.033***	0.679 (0.475, 0.970)	56.9%
Patients	9 (0.092)	51 (0.520)	38 (0.388)	*χ* ^2^ = 5.679	69 (0.352)	127 (0.648)			
Controls	37 (0.196)	94 (0.497)	58 (0.307)	*P* = 0.058	168 (0.444)	210 (0.556)			
rs3767546	A/A	A/T	T/T		A	T	0.457	0.751 (0.353, 1.597)	10.6%
Patients	0 (0)	10 (0.102)	89 (0.908)	*χ* ^2^ = 0.816	10 (0.051)	188 (0.949)			
Controls	1 (0.005)	23 (0.122)	165 (0.873)	*P* = 0.665	25 (0.067)	353 (0.934)			
rs117962659	C/C	C/A	A/A		C	A	0.186	1.920 (0.730, 5.049)	12.4%
Patients	0 (0)	6 (0.061)	93 (0.939)	*χ* ^2^ = 0.895	6 (0.030)	192 (0.970)			
Controls	1 (0.005)	15 (0.080)	172 (0.915)	*P* = 0.639	17 (0.045)	359 (0.955)			
rs2275860	A/A	A/G	G/G		A	G	0.448	0.745 (0.351, 1.588)	10.8%
Patients	1 (0.010)	8 (0.081)	90 (0.909)	*χ* ^2^ = 3.564	10 (0.051)	188 (0.949)			
Controls	0 (0)	25 (0.133)	163 (0.867)	*P* = 0.168	25 (0.066)	351 (0.934)			
c.192 + 353 C/A	C/C	C/A	A/A		C	A	***0.022***	0.095 (0.013, 0.714)	87.2%
Patients	98 (0.990)	1 (0.010)	0 (0)	*χ* ^2^ = 8.333	197 (0.995)	1 (0.005)			
Controls	168 (0.898)	19 (0.102)	0 (0)	***P* = 0.004**	355 (0.949)	19 (0.051)			
c.192 + 463 G/A	G/G	A/G	A/A		G	A	***0.019***	0.090 (0.012, 0.675)	89.7%
Patients	98 (0.99)	1 (0.01)	0 (0)	*χ* ^2^ = 8.925	197 (0.995)	1 (0.005)			
Controls	167 (0.893)	20 (0.107)	0 (0)	***P* = 0.003**	354 (0.947)	20 (0.053)			

Note: OR: odds ratio, at which the minor allele was viewed as an exposure factor in this case-control study.
